# Assessment of Nutritional Status of Children With Congenital Heart Disease: A Comparative Study

**DOI:** 10.3389/fnut.2021.644030

**Published:** 2021-09-17

**Authors:** Awoere T. Chinawa, Josephat M. Chinawa, Chika Onyinyechi Duru, Bartholomew F. Chukwu, Ijeoma Obumneme-Anyim

**Affiliations:** ^1^Department of Community Medicine, Enugu State University of Science and Technology, Enugu, Nigeria; ^2^Department of Pediatrics, College of Medicine, University of Nigeria, Enugu, Nigeria; ^3^Department of Pediatrics, Nigeria, Niger Delta University, Amassama, Nigeria

**Keywords:** children, congenital heart disease, nutritional status, malnutrition, anthro software

## Abstract

**Background:** Malnutrition poses a great burden to children in the tropics. However, this seems to be accentuated in children with congenital heart disease.

**Objectives:** The present study is therefore aimed at determining the nutritional status of children with congenital heart disease and to compare them with those without congenital heart disease.

**Methods:** This is a cross-sectional study, where congenital heart disease was diagnosed by means of echocardiograph. Anthro software was used to calculate Z scores for weight for age (WAZ), height for age (HAZ), and weight for height (WHZ). Body mass index (BMI) was calculated by the formula BMI = Weight (Kg)/height (M2).

**Results:** The body mass index-for-age z-score (BAZ) and height/length-for-age z-score (HAZ) were calculated for both subjects and controls to determine their nutritional status. It was observed that 38.5% (112/291) of the subjects were wasted (BAZ < −2SD) compared to 6.25% (16/256) of the controls and the difference was statistically significant (χ2 = 81.2, *p* < 0.001). Stunting (height/length-for-age z-score < −2SD) was also observed in a greater proportion of subjects than controls as 37.8% (107/291) of subjects were stunted compared with 7.0% (18/256) of the controls (χ2 = 69.9, *p* < 0.001). The under-five subjects had more cases of malnutrition than the controls of same age group as illustrated in Table 6. Whereas 42.9% (96/224) of the under-five subjects were wasted, only 6.2% (12/192) of the controls were wasted. On the other hand, 4.2% (8/192) of the under-five controls were obese compared to 0.9% (2/224) of the subjects of similar age group.

**Conclusion:** Children with congenital heart disease present with varying degrees of malnutrition that is worse compared with children without congenital heart disease. The impact of malnutrition is worse among children under the age of five. Wasting is more prevalent in children with cyanotic heart disease compared with those with acyanotic congenital heart disease. Overweight and obesity were notable features of malnutrition in children with congenital heart disease, but this is worse in children without congenital heart disease.

## Introduction

Congenital heart diseases are those diseases that occur at birth from abnormalities of cardiac structure due to aberration in development. (1) The rising trend in the prevalence of congenital heart disease is variable. Denise et al. (2) in a meta-analysis noted an increase in world prevalence from 0.6 per 1,000 live births in 1930 to 9.1 per 1,000 live births presently. Chinawa et al., (3) in Enugu, Nigeria, noted a prevalence of 0.22% among 31,795 children that attended children outpatient clinics. Congenital heart disease has been a serious challenge to affected families and relations. This is as a result of a complex interplay between high medical bills, cost of surgery, and heavy nutritional burden (4). There is a high burden of frequent hospital admission, poor surgical outcome, and death caused by malnutrition in children with congenital heart disease (4).

Adequate nutrition enhances physical and mental development, speeds academic performance, and helps in general well-being of a child from birth to adulthood. Infants and children are more likely to suffer from chronic malnutrition than older children (5, 6). Malnutrition afflicts children with congenital heart disease irrespective of presence or absence of cyanosis (7). Inadequate intake, increase basal metabolic rate from haemodynamic changes arising from the cardiac defects, and hypoxia have all been attributed to the cause of malnutrition in these children (8).

Malnutrition causes loss of body mass in children with congenital heart disease, especially the heart muscles. This in a long run will impair myocardial and pulmonary function of the heart. It also affects both cellular and humoral immunity with the consequent increase in risk of recurrent infection (9).

There are many studies on the assessment of nutritional status in children with congenital heart disease but studies that compare nutritional status with children without congenital heart disease are very few. This comparative study will help to know the actual burden of malnutrition in these children when compared to controls.

Some studies on nutritional status of children have been documented. For instance, findings on malnutrition in children with congenital heart disease in a particular study did not compare their findings with control (10). Furthermore, in the said study, malnutrition was classified as mild to severe without addressing the Z scores or BMI of these children, which will help to know whether nutritional derangement is of acute or chronic onset.

The present study is therefore aimed at determining the nutritional status of children with congenital heart disease and to compare them with those without congenital heart disease. It also aims to determine different degrees of malnutrition in subjects and controls.

## Methods

### Study Design

This was a comparative and a cross-sectional study that assessed the pattern of nutritional status among children with congenital heart disease in Enugu **over a five-year period (from 2016 to 2020)**. Children with congenital heart disease who attended the cardiology clinic and who fulfilled the inclusion criteria were consecutively recruited into the study. The controls were apparently healthy children who had no congenital or any cardiac disease, matched for age and sex.

### Anthropometry

Weight is measured with a weighing scale (sensitivity of 0.1 kilograms) while height was measure with stadiometer (floor type model with sensitivity of 0.1 cm): This instrument was used to measure the height of younger children and adolescents. It typically consists of a vertical ruler with a sliding horizontal rod or paddle which is adjusted to rest on top of the head. Infantometer (floor type model with sensitivity of 0.1 cm) was used to measure the length of infants. It is made of a sleek broad base with one sliding scale which is adjustable.

### Assessment of Nutritional Status

The World Health Organization (WHO) Anthro software was used to calculate Z scores for weight for age (WAZ), height for age (HAZ), and weight for height (WHZ).

The BMI was calculated by the formula BMI = Weight (Kg)/height (M2). The World Health Organization and Centers for Disease Control method was used to classify BMI into underweight as BMI less than the 5th percentile, healthy weight as BMI of 5th up to the 85th percentile, overweight as BMI of 85th to less than the 95th percentile, and obese as BMI equal to or greater than the 95th percentile for age and gender (11). Surface area was calculated using Mostellers formula (11) BSA (m^2^) = (height (cm) x weight (kg)/3600) ^½^.

### Settings

This study was carried out in four hospitals, the University of Nigeria Teaching Hospital (UNTH), Ituku-Ozalla, Enugu, Nigeria, Niger Delta University Bayelsa, Blessed children Hospital Enugu, and Triple Care Hospital Enugu. The University of Nigeria Teaching Hospital is a referral center for children with congenital heart disease all over the country and the South East region in particular, while Niger Delta University Bayelsa serves as a referral center for those in the South-South region. The other two hospitals serve as outlets where children with congenital heart disease were seen.

### Participants

These were children aged 1 week to 22 years who attended the cardiac clinic of the hospital under study. The control population were apparently healthy children matched for age and gender.

### Consent and Assent

This was obtained from each parent/caregiver of the subjects and controls while the latter was obtained from children older than seven years.

**Subjects with unrepaired cases of congenital heart defect (this includes both acyanotic and cyanotic congenital heart disease such as ASD, atrioventricular defect; VSD, ventricular septal defect; PDA, patent ductus arteriosus; TOF, tetralogy of Fallot; TGA, transposition of great arteries)** aged 1 week and 22 years were included into the study with their age and sex match controls while subjects and control whose parents refused to give consent were excluded.

**Study tool:** Echocardiography was performed in children with congenital heart disease using the Hewlett-Packard (HP) model SONO 2000 Ultrasound Imaging System. It has a probe with a frequency of between 5.5 and 12 MHz.

### Statistical Analysis

Categorical variables including gender and nutritional status were analyzed in the form of proportions and percentages and presented in the form of tables while discrete variables such as age and weight were summarized in the form of means and standard deviations. The z-scores of weight for height, weight-for-age, height/length-for-age, and body mass index (BMI) were calculated with WHO anthro and anthro plus software. The nutritional status of the children was based on WHO classification. Weight-for-height/length z-score (WHZ) was calculated for children under the age of five years while body mass index-for-age z-score (BAZ) and height/length-for-age (HAZ) was calculated for all age groups. Difference in proportions was compared with chi-square test while difference in means was compared with Student *t*-test. Significant level was set at *p* < 0.05.

## Result

Five hundred and forty-seven children participated in the study, comprising 291 children with various congenital heart diseases (subjects) and 256 children without congenital heart disease (controls). Table 1 depicts the gender distribution of the subjects and controls. The gender of subjects and controls were matched (χ2 = 0.57, *p* = 0.45). The mean age in months of the subjects, 43.1 (51.7), and the controls, 49.5 (36.1), were matched as well (Student *t-*test = −1.7, *p* = 0.1). Among the subjects, 78.4% (228/291) had acyanotic congenital heart disease and 21.6% (63/291) had cyanotic congenital heart disease.

**Table 1:** Gender distribution of the subjects and controls.

		**Gender**		**Total (%)**
		**Male (%)**	**Female (%)**	
	Subjects	152 (52.2)	139 (47.8)	291 (100.0)
	Controls	142 (55.5)	114 (44.5)	256 (100.0)
Total		266 (52.8)	238 (47.2)	547 (100.0)

The age group distribution of the subjects and controls is shown in Table 2, indicating that the majority of the participants are under the age of five years and the distribution was similar in both groups (χ2 = 4.9, *p* = 0.1).

**Table 2:** Age group distribution of subjects and controls.

		**Age group**			**Total (%)**
		**Under five (%)**	**Child (%)**	**Adolescent (%)**	
	Subjects	224 (77.0)	36 (12.4)	31 (10.6)	291 (100.0)
	Controls	192 (75.0)	46 (18.0)	18 (7.0)	256 (100.0)
Total		416 (76.1)	82 (15.0)	49 (8.9)	547 (100.0)

Under five (1 to 60 months), child (61 to 120 months), adolescent (121 to 216 months).

The mean weight, height/length, and body mass index (BMI) of subjects were significantly lower than that of the controls as illustrated in Table 3.

**Table 3:** Mean weight (Kg), height/length (cm), and BMI (Kg/M^2^) of subjects and controls.

	**Group**	**N**	**Mean**	**Std. Deviation**	***t***	***P***
Weight (kg)	Subjects	291	13.72	13.0		
	Controls	256	17.48	8.8	−3.89	<0.001
Height (cm)	Subjects	291	89.07	31.3		
	Controls	256	101.57	23.8	−5.21	<0.001
BMI (kg/m^2^)	Subjects	291	14.72	3.3		
	Controls	256	15.96	2.5	−4.91	<0.001

Among the subjects, the mean weight was comparable between those with cyanotic, 13.3 (8.1) kg, and acyanotic, 13.8 (14.1) kg, congenital heart disease (*t* = 0.3, *p* = 0.8). The BMI was also comparable between those with cyanotic congenital heart disease, 14.1 (2.5) kg/m^2^, and those with acyanotic congenital heart defects, 14.8 (3.4) kg/m^2^ (*t* = 1.7, *p* = 0.1). On the other hand, those with cyanotic defect had higher mean height/length than those with acyanotic defects, 96.9 (27.1) vs. 86.9 (32.0) cm (*t* = −2.3, *p* = 0.02).

Among the subjects, the mean weight was significantly higher in females, 15.4 (14.2) kg, than in males, 12.2 (11.7) kg, {*t* = −2.1, *p* = 0.04}. The mean height/length in females, 93.5 (32.5) cm, was also significantly higher than that of males, 85.0 (29.7) cm, {*t* = −2.3, *p* = 0.02}. However, the mean BMI in females, 15.9 (2.7) kg/m^2^, was comparable to that of the males, 16.0 (2.3) kg/m^2^, {*t* = 0.1, 0.9}.

The nutritional status of subjects and controls is as in Tables 4, 5. The body mass index-for-age z-score (BAZ) and height/length-for-age z-score (HAZ) were calculated for both subjects and controls to determine their nutritional status. It was observed that 38.5% (112/291) of the subjects were wasted (BAZ < −2SD) compared to 6.25% (16/256) of the controls and the difference was statistically significant (χ2 = 81.2, *p* < 0.001). Stunting (height/length-for-age z-score < −2SD) was also observed in a greater proportion of subjects than controls as 37.8% (107/291) of subjects were stunted compared with 7.0% (18/256) of the controls (χ2 = 69.9, *p* < 0.001).

**Table 4:** Nutritional status of subjects and controls based on BAZ.

		**Nutritional status**				**Total**
		**Normal *n* (%)**	**Wasted*n* (%)**	**Overweight *n* (%)**	**Obese*n* (%)**	
	Subjects	164 (56.4)	112 (38.5)	11 (3.8)	4 (1.4)	291 (100.0)
	Controls	209 (81.6)	16 (6.3)	21 (8.2)	10 (3.9)	256 (100.0)
Total		373 (68.2)	128 (23.4)	32 (5.9)	14 (2.6)	547 (100.0)

*BAZ, Body mass index-for-age z-score. χ2 = 81.2, p < 0.001*.

**Table 5:** Nutritional status of participants based on HAZ.

		**Nutritional status**			**Total**
		**Normal *n* (%)**	**Stunted*n* (%)**	**Severely stunted *n* (%)**	
	Subjects	184 (63.2)	41 (14.1)	66 (22.7)	291 (100.0)
	Controls	238 (93.0)	11 (4.3)	7 (2.7)	256 (100.0)
Total		422 (77.1)	52 (9.5)	73 (13.3)	547 (100.0)

*HAZ, height/length-for-age z-score. χ2 = 69.9, p < 0.001*.

The under-five subjects had more cases of malnutrition than the controls of same age group as illustrated in Table 6. Whereas 42.9% (96/224) of the under-five subjects were wasted, only 6.2% (12/192) of the controls were wasted. On the other hand, 4.2% (8/192) of the under-five controls were obese compared to 0.9% (2/224) of the subjects of similar age group.

**Table 6:** Nutritional status of different age groups based on BAZ.

			**Nutritional status**				**Total*N* (%)**
			**Normal *n* (%)**	**Wasted*n* (%)**	**Overweight *n* (%)**	**Obese*n* (%)**	
Subjects	Age group	Under five	119 (53.1)	96 (42.9)	7 (3.1)	2 (0.9)	224 (100.0)
		Child	21 (58.3)	11 (30.5)	2 (5.6)	2 (5.6)	36 (100.0)
		Adolescent	24 (77.4)	5 (16.1)	2 (6.5)	0 (0)	31 (100.0)
	Total		164 (56.3)	112 (38.5)	11 (3.8)	4 (1.4)	291 (100.0)
Controls	Age group	Under five	153 (79.7)	12 (6.2)	19 (9.9)	8 (4.2)	192 (100.0)
		Child	40 (86.9)	3 (6.5)	1 (2.2)	2 (4.3)	46 (100.0)
		Adolescent	16 (89.0)	1 (5.5)	1 (5.5)	0 (0)	18 (100.0)
	Total		209 (81.6)	16 (6.2)	21 (8.2)	10 (4.0)	256 (100.0)

*BAZ, body mass index-for-age z-score*.

Also, 16.5% (37/224) of subjects compared with 4.7% (9/192) of controls of the under-five age group were stunted whereas 26.3% and 3.6% had severe stunting (HAZ, −3SD) respectively as shown in Table 7. The difference in proportion of under-five children with stunting between the subjects and control was statistically significant (χ2 = 15.7, *p* = 0.003).

**Table 7:** Nutritional status of subjects and controls based on HAZ.

			**Nutritional status**			**Total**
			**Normal *n* (%)**	**Stunted*n* (%)**	**Severely stunted *n* (%)**	
Subjects	Age group	Under five	128 (57.1)	37 (16.5)	59 (26.3)	224 (100.0)
		Child	31 (86.1)	2 (5.6)	3 (8.3)	36 (100.0)
		Adolescent	25 (80.6)	2 (6.4)	4 (13.0)	31 (100.0)
	Total		184 (63.2)	41 (14.1)	66 (26.7)	291 (100.0)
Controls	Age group	Under five	176 (91.7)	9 (4.7)	7 (3.6)	192 (100.0)
		Child	45 (97.8)	1 (2.2)	0 (0)	46 (100.0)
		Adolescent	17 (94.4)	1 (5.6)	0 (0)	18 (100.0)
	Total		238 (93.0)	11 (4.3)	7 (2.7)	256 (100.0)

*HAZ, height/length-for-age z-score*.

Among the subjects, those with cyanotic congenital heart disease had relatively higher cases of malnutrition than those with acyanotic disease. A total of 42.8% (27/63) of those cyanotic disease were wasted compared with 37.3% (85/228) of those with acyanotic disease. However, the difference was not statistically significant (χ2 = 2.54, *p* = 0.5).

## Discussion

This study showed that 38.5% of children with congenital heart disease were wasted compared to 6.25% of the controls, while stunting was also observed in 37.8% compared with 7.0% seen in children without congenital heart disease. This finding was lower when compared with that of Basheir et al. (12) who noted a high prevalence of 84%. Higher prevalence has also been documented in several studies compared to ours. For instance, rates of 85%, 59%, and 90.4% have been reported in Turkey, (13) South India, (14) and Nigeria, (15) respectively. Methodology and large sample size could explain the lower prevalence seen in this study.

However, Batte et al. (16) also noted similar prevalence of wasting and stunting as 31.5 and 45.4% respectively, while Okoroma et al. (15) noted wasting and stunting as 41.1, 28.8, 2.6, and 3.9% in subjects and controls. There is a decreasing trend of malnutrition in children with congenital heart disease when compared to what was obtained some years ago. Advanced knowledge in nutritional rehabilitation, increased awareness, and frequency in the diagnosis of congenital heart disease could explain this declining trend.

Children with cyanotic congenital heart disease present with more episodes of wasting than their acyanotic counterpart. This study is also in keeping with that of Basheir et al. (12) who noted that stunting was associated with acyanotic CHD, and wasting with cyanotic CHD. Several studies have reported different findings in this regard. For instance, Okoromah and his colleagues (15) reported wasting more in left to right shunt lesions. Chronic hypoxia from right to left lesion with possible prolonged pulmonary hypertension seen in the cyanotic heart disease could explain these differences.

The mean weight and height/length was significantly higher in females with congenital heart disease. Female children have small body surface area at birth compared to their male counterparts. This issue couple with genetic variation could also explain these differences in nutritional status (17–19).

Children with congenital heart disease who are under the age of five are more prone to malnutrition than the controls of the same age group. In our setting, surgical intervention occurs very late in these children. Several studies have shown that delayed surgical correction of congenital heart disease is associated with malnutrition which is worse in children less than five years (15, 19–25). Furthermore progressive hypoxia, worsening pulmonary hypertension, and shunt reversal (harbingers of malnutrition) are common sequels that start in children less than five years old who had congenital heart disease with no intervention.

It is interesting to note from this study that children with cyanotic congenital heart defect had higher mean height/length than those with acyanotic congenital heart disease. Increased metabolic stress with resultant release of free oxygen radicals, ischemia, reperfusion, and chronic hypoxia seen commonly in children with cyanotic congenital heart disease could explain this difference (26).

Overweight and obesity were noted in 3.8 and 1.4% of children with congenital heart disease respectively, compared with 8.2 and 3.8% seen in controls. In the United States, a prevalence of over 25% of obese and overweight children were documented among children with congenital heart disease (27). This was lower in a study in Belgium where a prevalence of 7.6% was obtained. However, the study in Belgium is basically among adolescents (28).

Restrictions of physical activity and excessive nutritional rehabilitation are used for weight gain especially for infancy (29) which includes consumption of increased calories and foods with high fat and sodium content (30, 31). There had been increased risk of mal- and undernutrition in children with congenital heart disease which stems from increased metabolic rates, malabsorption, hypoxia and pulmonary hypertension (32). Most intervention therefore focused on treatment strategies that prioritize adequate growth and development. These strategies include increased caloric intake not considering the type and severity of the cardiac lesion (21, 33).

We used body mass index-for-age z-score (BAZ) and height/length-for-age z-score (HAZ) in assessing nutritional status in this study. Height/length-for-age z-score (HAZ) is used in the diagnosis of stunting while the BMI is used for overweight and obesity (22, 23).

It is important to stress here that there had been lots of screening tools for nutritional status including the use of BMI (27, 28). BMI is very useful for population studies and epidemiological studies (34–36).

## Conclusion

Children with congenital heart disease present with varying degrees of malnutrition that is worse compared with children without congenital heart disease. The impact of malnutrition is worse among children under the age of five. Wasting is more prevalent in children with cyanotic heart disease compared with those with acyanotic congenital heart disease. Overweight and obesity were notable features of malnutrition in children with congenital heart disease but this is worse in children without congenital heart disease.

## Data Availability Statement

The raw data supporting the conclusions of this article will be made available by the authors, without undue reservation.

## Ethics Statement

The studies involving human participants were reviewed and approved by UNIVERSITY OF NIGERIA. Written informed consent to participate in this study was provided by the participants' legal guardian/next of kin.

## Author Contributions

JMC contributed to the conception, while JMC, ATC, COD, BFC, and IO-A contributed in writing and proof reading of this manuscript. JMC and BFC are guarantors of the paper. All authors read and approved the final manuscript.

## Conflict of Interest

The authors declare that the research was conducted in the absence of any commercial or financial relationships that could be construed as a potential conflict of interest.

## Publisher's Note

All claims expressed in this article are solely those of the authors and do not necessarily represent those of their affiliated organizations, or those of the publisher, the editors and the reviewers. Any product that may be evaluated in this article, or claim that may be made by its manufacturer, is not guaranteed or endorsed by the publisher.
